# Project ECHO in Geriatrics Education: Innovative Interprofessional Models and Methods for the Future

**DOI:** 10.1093/geroni/igaf122.746

**Published:** 2025-12-31

**Authors:** Phillip Clark, Thomas Caprio

## Abstract

Future innovation in geriatrics education depends on integrating interprofessional teamwork with Age-Friendly 4M Care principles in targeted settings and with specific types of trainees. The 4M framework requires coordinated participation of multiple professions to address the multifaceted and complex healthcare needs of older adults. Project ECHO (Extension for Community Healthcare Outcomes) is custom-made for this purpose. It is a Zoom-based telemedicine model with an interprofessional hub of specialists delivering sessions to primary care and other teams in the community. Key components are a short didactic presentation, followed by a case presentation and discussion. An “all teach, all learn” educational philosophy levels professional hierarchies, with participants sharing perspectives and recommendations for a complex case in a community of practice built on mutual support and trust. Papers in this symposium are based on the extensive ECHO experience of several HRSA-funded Geriatric Workforce Enhancement Programs (GWEPs) and focus on key lessons learned from specific settings and trainees for expanding future ECHOs. First, the conceptual basis of ECHO for the development of interprofessional education (IPE) is presented. Second, the implications of lessons learned from multi-GWEP collaboration in designing Dementia-Friendly education for primary care teams are presented. Third, the challenges of fostering teamwork with front-line CNA providers are explored. Finally, methods of combining IPE and geriatrics education of students and other trainees are explored. Recommendations include developing more explicit methods for integrating 4M care and teamwork, targeting ECHOs on specific clinical teams and sites, and more effectively assessing impacts on learners and clinical practice.

